# 1,2:5,6-Di-*O*-iso­propyl­idene-3-*C*-methyl-α-d-allo­furan­ose

**DOI:** 10.1107/S1600536813022447

**Published:** 2013-09-07

**Authors:** Luana da Silva Magalhães Forezi, Marcos Moitrel Pequeno Silva, Fernanda da Costa Santos, Vitor Francisco Ferreira, Maria Cecília Bastos Vieira de Souza

**Affiliations:** a Departamento de Química Orgânica, Instituto de Química, Universidade Federal Fluminense, Niterói – RJ, CEP 24020-150, Brazil; b Departamento de Química Inorgânica, Instituto de Química, Universidade Federal Fluminense, Niterói – RJ, CEP 24020-150, Brazil

## Abstract

The title carbohydrate, C_13_H_22_O_6_, is a derivative of d-glycose, in which the furan­osidic and iso­propyl­idene rings are in twisted conformations. The mean plane of the furan­osidic ring makes a dihedral angle of 70.32 (18)° with the mean plane of the fused iso­propyl­idene ring. The methyl groups in the other iso­propyl­idene ring are disordered over two sets of sites, with an occupancy ratio of 0.74 (6):0.26 (6). In the crystal, mol­ecules are linked by O—H⋯O hydrogen bonds into chains with graph-set notation *C*(5) along [100]. Weak C—H⋯O interactions also occur.

## Related literature

For background information on this class of compound, see: Bio *et al.* (2004 ▶); Canuto *et al.* (2007 ▶); Mane *et al.* (2008 ▶); Yoneda *et al.* (2011 ▶). For details of ring-puckering calculations, see: Cremer & Pople (1975 ▶). Graph-set notation for the description of hydrogen-bonding motifs is given by Bernstein *et al.* (1995 ▶).
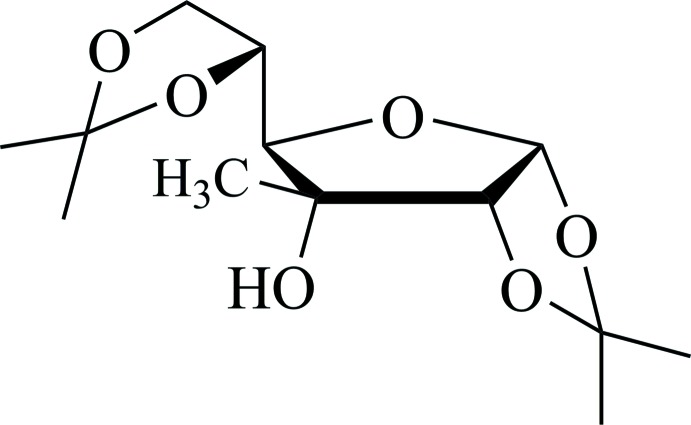


## Experimental

### 

#### Crystal data

C_13_H_22_O_6_
*M* *_r_* = 274.3
Triclinic, 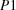

*a* = 5.503 (4) Å
*b* = 8.113 (1) Å
*c* = 9.122 (2) Å
α = 99.65 (2)°
β = 103.69 (3)°
γ = 98.86 (2)°
*V* = 382.0 (3) Å^3^
*Z* = 1
Mo *K*α radiation
μ = 0.09 mm^−1^
*T* = 293 K
0.14 × 0.11 × 0.08 mm

#### Data collection

Nonius KappaCCD diffractometer
9589 measured reflections
2758 independent reflections
2107 reflections with *I* > 2σ(*I*)
*R*_int_ = 0.043

#### Refinement

*R*[*F* ^2^ > 2σ(*F* ^2^)] = 0.046
*wR*(*F* ^2^) = 0.130
*S* = 1.04
2758 reflections
181 parameters
8 restraints
H-atom parameters constrained
Δρ_max_ = 0.21 e Å^−3^
Δρ_min_ = −0.24 e Å^−3^
Absolute structure: Flack *x* calculated using 872 quotients [(*I* ^+^) − (*I* ^−^)]/[(*I* ^+^) + (*I* ^−^)] (Parsons & Flack, 2004 ▶). There is insufficient information present to define handedness

### 

Data collection: *COLLECT* (Nonius, 2004 ▶); cell refinement: *DIRAX/LSQ* (Duisenberg, 1992 ▶); data reduction: *EVALCCD* (Duisenberg *et al.*, 2003 ▶); program(s) used to solve structure: *SHELXS97* (Sheldrick, 2008 ▶); program(s) used to refine structure: *SHELXL2013* (Sheldrick, 2013 ▶); molecular graphics: *ORTEP-3 for Windows* (Farrugia, 2012 ▶); software used to prepare material for publication: *WinGX* (Farrugia, 2012 ▶).

## Supplementary Material

Crystal structure: contains datablock(s) global, I. DOI: 10.1107/S1600536813022447/pk2484sup1.cif

Structure factors: contains datablock(s) I. DOI: 10.1107/S1600536813022447/pk2484Isup2.hkl

Additional supplementary materials:  crystallographic information; 3D view; checkCIF report

## Figures and Tables

**Table 1:** Hydrogen-bond geometry (Å, °)

*D*—H⋯*A*	*D*—H	H⋯*A*	*D*⋯*A*	*D*—H⋯*A*
O3—H3⋯O4^i^	0.82	2.28	3.022 (4)	152
C1—H1⋯O2^ii^	0.98	2.58	3.218 (5)	123
C2—H2⋯O6^iii^	0.98	2.54	3.504 (5)	167

Symmetry codes: (i) 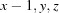; (ii) 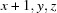; (iii) 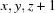.

**Fig. 1.**
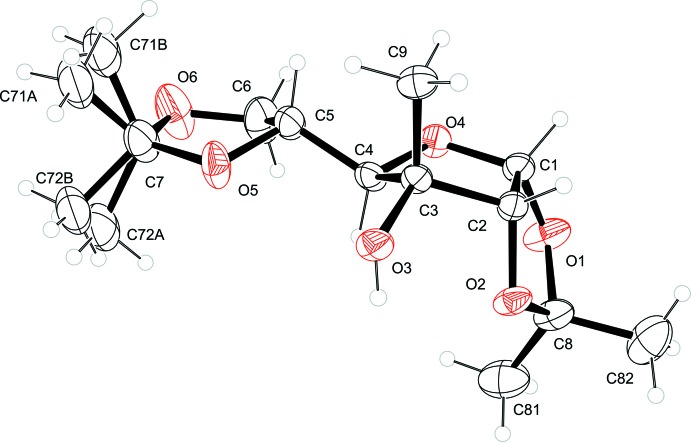
Ellipsoid plot representation of the molecular structure of compound I with displacement ellipsoids drawn at the 30% probability level. Atoms C71A/B and C72A/B are disordered with fractional occupancies of 0.74 (6):0.26 (6) for the A and B components, respectively.

**Fig. 2.**
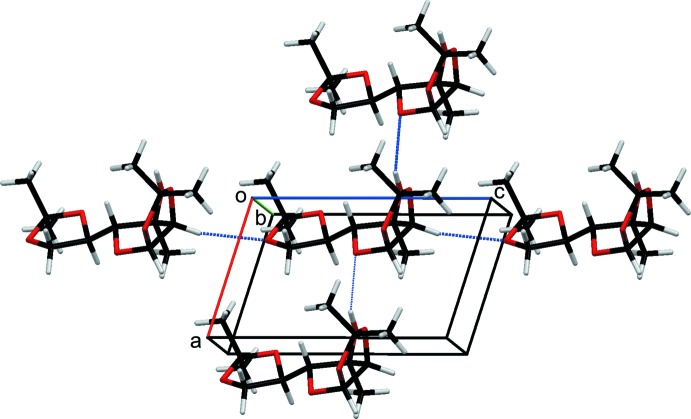
A packing diagram of (I), viewed approximately down the b axis. Hydrogen-bonds are shown by dashed lines.

## supplementary crystallographic information

### 1. Comment

The structural modification of carbohydrates has been extensively explored for improvement of their pharmacology properties (Bio *et al.*, 2004; Canuto *et al.* (2007); Mane *et al.* 2008; Yoneda *et al.* 2011). As ongoing research in developing potentially new drugs, we report here the structure of 1,2:5,6-Di-*O*-isopropylidene-3-*C*-methyl-α-*D*-allofuranose.

In the title molecule (Fig. 1), the torsion angle formed by atoms O4, C1, C2, O2 is 101.6 (3)° and that formed by O1, C1, C2, C3, is -130.8 (3)°.

The structure exhibits disorder in a isopropylidene group (atoms C71A, C72A, C71B and C72B) over two positions. Rings A (O1,C1,C2,O2,C8) and B (C1,C2,C3,C4,O4) adopt a twisted conformation, with ring-puckering parameters q2 = 0.223 (5) Å, φ2 = 277 (1)°; q2 = 0.376 (5) Å, φ2 = 89.8 (6)°, respectively. Ring C (O5,C5,C6,O6,C7) also shows a twist conformation, with ring-puckering parameters q2 = 0.404 (3) Å, φ2 = 196.021 (1)° (Cremer & Pople, 1975).

In the crystal packing, molecules are linked by O—H···O hydrogen bonds into chains with graph-set notation *C*(5) along [100] (Bernstein *et al.*, 1995). There are also short C—H···O interactions that form a *C*(7) chain motif along [001] direction (Fig. 2).

### 2. Experimental

The reaction for obtaining the title compound was taken under nitrogen in a tritubulate vessel. To it, 2.64 ml (3.95 mmol) of methyl magnesium bromide 3 *M* diluted in THF (7.9 mmol, 2 eq) was added. Then, under vigorous stirring and in a ice bath, 3.96 mmol (1.02 g) diluted in dry THF was poured into the solution. The reaction took place for 5 h in room temperature. After finishing the reaction, it was slowly added dropwise 10 ml of distilled water and 1 g of Celite, filtering the resultant product in a Celite layer. The THF solvent was removed by heat. The aqueous phase was extracted with CH_2_Cl_2_ (3 × 30 ml) and the product washed with distilled water (3 × 20 ml), dried with MgSO_4_ anhydrous and the solvent eliminated on a vacuum rotator evaporator apparatus. The solid was recrystallized in hexane and the yellow solid product was obtained with 72% yield. (m.p. = 104–105°C) (Bio *et al.*, 2004).

### 3. Refinement

The H atoms were placed at calculated idealized positions and refined using a riding model with individual displacement parameters *U*_iso_(H) = 1.2*U*_eq_ (C*sp*^2^) or 1.5*U*_eq_ (methyl and hydroxyl groups).

### Crystal data

**Table:**

C_13_H_22_O_6_	*Z* = 1
*M**_r_* = 274.3	*F*(000) = 148
Triclinic, *P*1	*D*_x_ = 1.192 Mg m^−^^3^
Hall symbol: P 1	Mo *K*α radiation, λ = 0.71073 Å
*a* = 5.503 (4) Å	Cell parameters from 3284 reflections
*b* = 8.113 (1) Å	θ = 3.1–27.5°
*c* = 9.122 (2) Å	µ = 0.09 mm^−^^1^
α = 99.65 (2)°	*T* = 293 K
β = 103.69 (3)°	Prism, colourless
γ = 98.86 (2)°	0.14 × 0.11 × 0.08 mm
*V* = 382.0 (3) Å^3^	

### Data collection

**Table:**

Nonius KappaCCD diffractometer	2107 reflections with *I* > 2σ(*I*)
Radiation source: Enraf–Nonius FR590	*R*_int_ = 0.043
Graphite monochromator	θ_max_ = 25.7°, θ_min_ = 3.1°
Detector resolution: 9 pixels mm^-1^	*h* = −6→6
CCD rotation images, thick slices scans	*k* = −9→9
9589 measured reflections	*l* = −11→11
2758 independent reflections	

### Refinement

**Table:**

Refinement on *F*^2^	H-atom parameters constrained
Least-squares matrix: full	*w* = 1/[σ^2^(*F*_o_^2^) + (0.0729*P*)^2^ + 0.0707*P*] where *P* = (*F*_o_^2^ + 2*F*_c_^2^)/3
*R*[*F*^2^ > 2σ(*F*^2^)] = 0.046	(Δ/σ)_max_ < 0.001
*wR*(*F*^2^) = 0.130	Δρ_max_ = 0.21 e Å^−^^3^
*S* = 1.04	Δρ_min_ = −0.24 e Å^−^^3^
2758 reflections	Extinction correction: *SHELXL2013* (Sheldrick, 2013)
181 parameters	Extinction coefficient: 0.20 (3)
8 restraints	Absolute structure: Flack *x* calculated using 872 quotients [(I+)-(I-)]/[(I+)+(I-)] (Parsons & Flack, 2004). There is insufficient information present to define handedness.
Hydrogen site location: inferred from neighbouring sites	Absolute structure parameter: −0.2 (5)

### Special details

**Table:**

Geometry. All e.s.d.'s (except the e.s.d. in the dihedral angle between two l.s. planes) are estimated using the full covariance matrix. The cell e.s.d.'s are taken into account individually in the estimation of e.s.d.'s in distances, angles and torsion angles; correlations between e.s.d.'s in cell parameters are only used when they are defined by crystal symmetry. An approximate (isotropic) treatment of cell e.s.d.'s is used for estimating e.s.d.'s involving l.s. planes.

### Fractional atomic coordinates and isotropic or equivalent isotropic displacement parameters (Å^2^)

**Table:**

	*x*	*y*	*z*	*U*_iso_*/*U*_eq_	Occ. (<1)
O1	0.2339 (6)	−0.0979 (4)	0.6028 (5)	0.0646 (10)	
O2	−0.0659 (5)	0.0519 (3)	0.6425 (3)	0.0456 (8)	
O3	−0.0994 (5)	0.3540 (4)	0.5720 (3)	0.0434 (7)	
H3	−0.2119	0.2677	0.5383	0.065*	
O4	0.3788 (5)	0.1260 (4)	0.4944 (3)	0.0446 (7)	
O5	0.0585 (6)	0.3883 (5)	0.2656 (4)	0.0633 (10)	
O6	0.2663 (8)	0.3148 (8)	0.0861 (5)	0.1016 (17)	
C1	0.3517 (7)	0.0751 (5)	0.6312 (5)	0.0394 (9)	
H1	0.5169	0.0992	0.7088	0.047*	
C2	0.1689 (7)	0.1724 (5)	0.6883 (4)	0.0357 (9)	
H2	0.2256	0.2197	0.7997	0.054*	
C3	0.1409 (6)	0.3087 (5)	0.5930 (4)	0.0327 (9)	
C4	0.1819 (7)	0.2156 (5)	0.4429 (5)	0.0397 (9)	
H4	0.0255	0.1324	0.3853	0.048*	
C5	0.2672 (8)	0.3188 (6)	0.3367 (5)	0.0486 (11)	
H5	0.4124	0.411	0.3948	0.058*	
C6	0.3295 (12)	0.2203 (10)	0.2017 (6)	0.0787 (18)	
H6A	0.5092	0.2151	0.2255	0.094*	
H6B	0.2282	0.1051	0.1701	0.094*	
C7	0.0949 (10)	0.4150 (9)	0.1214 (6)	0.0727 (17)	
C71A	0.222 (7)	0.598 (3)	0.144 (3)	0.149 (7)	0.74 (6)
H71A	0.0946	0.6662	0.1244	0.223*	0.74 (6)
H71C	0.3259	0.6077	0.0732	0.223*	0.74 (6)
H71B	0.3279	0.6382	0.2478	0.223*	0.74 (6)
C72A	−0.147 (3)	0.345 (4)	−0.0032 (17)	0.116 (6)	0.74 (6)
H72A	−0.1074	0.3085	−0.099	0.174*	0.74 (6)
H72B	−0.2456	0.4326	−0.0132	0.174*	0.74 (6)
H72C	−0.2427	0.2503	0.0222	0.174*	0.74 (6)
C71B	0.204 (18)	0.595 (8)	0.107 (11)	0.149 (7)	0.26 (6)
H71D	0.1085	0.6732	0.145	0.223*	0.26 (6)
H71E	0.1935	0.5954	0.0005	0.223*	0.26 (6)
H71F	0.3798	0.6279	0.1663	0.223*	0.26 (6)
C72B	−0.180 (6)	0.404 (11)	0.027 (6)	0.116 (6)	0.26 (6)
H72D	−0.1971	0.3568	−0.0794	0.174*	0.26 (6)
H72E	−0.2186	0.5155	0.0375	0.174*	0.26 (6)
H72F	−0.2952	0.3311	0.0647	0.174*	0.26 (6)
C8	−0.0148 (8)	−0.1149 (6)	0.6267 (6)	0.0517 (12)	
C81	−0.2032 (11)	−0.2263 (8)	0.4845 (8)	0.0820 (17)	
H81A	−0.1132	−0.2828	0.4198	0.123*	
H81B	−0.3017	−0.1573	0.429	0.123*	
H81C	−0.3152	−0.31	0.5137	0.123*	
C82	−0.0177 (13)	−0.1798 (8)	0.7692 (8)	0.0823 (18)	
H82A	0.1478	−0.2012	0.8139	0.123*	
H82C	−0.1428	−0.2839	0.7441	0.123*	
H82B	−0.0598	−0.0963	0.8418	0.123*	
C9	0.3413 (8)	0.4681 (5)	0.6694 (5)	0.0454 (10)	
H9C	0.3209	0.5524	0.6076	0.068*	
H9B	0.5079	0.4417	0.6796	0.068*	
H9A	0.3227	0.5117	0.7699	0.068*	

### Atomic displacement parameters (Å^2^)

**Table:**

	*U*^11^	*U*^22^	*U*^33^	*U*^12^	*U*^13^	*U*^23^
O1	0.0496 (18)	0.0411 (18)	0.120 (3)	0.0181 (14)	0.0450 (19)	0.0236 (18)
O2	0.0326 (14)	0.0361 (15)	0.079 (2)	0.0128 (12)	0.0242 (13)	0.0242 (14)
O3	0.0304 (14)	0.0420 (16)	0.0656 (19)	0.0149 (12)	0.0176 (13)	0.0193 (14)
O4	0.0368 (14)	0.0536 (17)	0.0531 (17)	0.0194 (13)	0.0218 (12)	0.0149 (14)
O5	0.066 (2)	0.102 (3)	0.0433 (17)	0.0418 (19)	0.0250 (15)	0.0373 (17)
O6	0.079 (3)	0.203 (5)	0.059 (2)	0.070 (3)	0.039 (2)	0.056 (3)
C1	0.031 (2)	0.043 (2)	0.052 (2)	0.0133 (17)	0.0156 (17)	0.0175 (19)
C2	0.0308 (19)	0.041 (2)	0.040 (2)	0.0105 (16)	0.0139 (16)	0.0117 (17)
C3	0.0270 (18)	0.037 (2)	0.038 (2)	0.0114 (16)	0.0119 (15)	0.0107 (17)
C4	0.0302 (19)	0.048 (2)	0.041 (2)	0.0087 (17)	0.0079 (16)	0.0101 (18)
C5	0.039 (2)	0.070 (3)	0.042 (2)	0.014 (2)	0.0138 (19)	0.021 (2)
C6	0.078 (4)	0.122 (5)	0.061 (3)	0.045 (4)	0.038 (3)	0.037 (3)
C7	0.055 (3)	0.132 (5)	0.045 (3)	0.030 (3)	0.019 (2)	0.037 (3)
C71A	0.239 (13)	0.140 (8)	0.076 (15)	0.001 (8)	0.059 (11)	0.059 (8)
C72A	0.057 (5)	0.26 (2)	0.048 (6)	0.069 (7)	0.023 (4)	0.033 (8)
C71B	0.239 (13)	0.140 (8)	0.076 (15)	0.001 (8)	0.059 (11)	0.059 (8)
C72B	0.057 (5)	0.26 (2)	0.048 (6)	0.069 (7)	0.023 (4)	0.033 (8)
C8	0.043 (2)	0.041 (2)	0.083 (3)	0.017 (2)	0.031 (2)	0.021 (2)
C81	0.066 (4)	0.056 (3)	0.115 (5)	0.002 (3)	0.025 (3)	0.005 (3)
C82	0.097 (4)	0.073 (4)	0.112 (5)	0.041 (3)	0.055 (4)	0.056 (4)
C9	0.043 (2)	0.038 (2)	0.056 (2)	0.0101 (18)	0.0146 (18)	0.0080 (18)

### Geometric parameters (Å, º)

**Table:**

O1—C1	1.405 (5)	C7—C71B	1.53 (3)
O1—C8	1.426 (5)	C7—C72B	1.53 (2)
O2—C8	1.415 (5)	C71A—H71A	0.96
O2—C2	1.421 (5)	C71A—H71C	0.96
O3—C3	1.405 (4)	C71A—H71B	0.96
O3—H3	0.82	C72A—H72A	0.96
O4—C1	1.412 (5)	C72A—H72B	0.96
O4—C4	1.428 (5)	C72A—H72C	0.96
O5—C7	1.422 (6)	C71B—H71D	0.96
O5—C5	1.423 (5)	C71B—H71E	0.96
O6—C7	1.395 (8)	C71B—H71F	0.96
O6—C6	1.415 (8)	C72B—H72D	0.96
C1—C2	1.504 (5)	C72B—H72E	0.96
C1—H1	0.98	C72B—H72F	0.96
C2—C3	1.521 (5)	C8—C82	1.485 (8)
C2—H2	0.98	C8—C81	1.503 (8)
C3—C9	1.506 (6)	C81—H81A	0.96
C3—C4	1.532 (6)	C81—H81B	0.96
C4—C5	1.493 (5)	C81—H81C	0.96
C4—H4	0.98	C82—H82A	0.96
C5—C6	1.492 (7)	C82—H82C	0.96
C5—H5	0.98	C82—H82B	0.96
C6—H6A	0.97	C9—H9C	0.96
C6—H6B	0.97	C9—H9B	0.96
C7—C72A	1.489 (12)	C9—H9A	0.96
C7—C71A	1.502 (16)		
			
C1—O1—C8	110.7 (3)	O5—C7—C72B	102 (2)
C8—O2—C2	109.1 (3)	C71B—C7—C72B	97 (2)
C3—O3—H3	109.5	C7—C71A—H71A	109.5
C1—O4—C4	109.0 (3)	C7—C71A—H71C	109.5
C7—O5—C5	106.9 (4)	H71A—C71A—H71C	109.5
C7—O6—C6	109.5 (4)	C7—C71A—H71B	109.5
O1—C1—O4	111.8 (4)	H71A—C71A—H71B	109.5
O1—C1—C2	105.2 (3)	H71C—C71A—H71B	109.5
O4—C1—C2	106.8 (3)	C7—C72A—H72A	109.5
O1—C1—H1	110.9	C7—C72A—H72B	109.5
O4—C1—H1	110.9	H72A—C72A—H72B	109.5
C2—C1—H1	110.9	C7—C72A—H72C	109.5
O2—C2—C1	104.0 (3)	H72A—C72A—H72C	109.5
O2—C2—C3	108.0 (3)	H72B—C72A—H72C	109.5
C1—C2—C3	105.1 (3)	C7—C71B—H71D	109.5
O2—C2—H2	113	C7—C71B—H71E	109.5
C1—C2—H2	113	H71D—C71B—H71E	109.5
C3—C2—H2	113	C7—C71B—H71F	109.5
O3—C3—C9	107.8 (3)	H71D—C71B—H71F	109.5
O3—C3—C2	112.6 (3)	H71E—C71B—H71F	109.5
C9—C3—C2	110.7 (3)	C7—C72B—H72D	109.5
O3—C3—C4	112.9 (3)	C7—C72B—H72E	109.5
C9—C3—C4	112.8 (3)	H72D—C72B—H72E	109.5
C2—C3—C4	100.0 (3)	C7—C72B—H72F	109.5
O4—C4—C5	107.2 (3)	H72D—C72B—H72F	109.5
O4—C4—C3	103.8 (3)	H72E—C72B—H72F	109.5
C5—C4—C3	118.7 (3)	O2—C8—O1	105.1 (3)
O4—C4—H4	108.9	O2—C8—C82	110.5 (4)
C5—C4—H4	108.9	O1—C8—C82	110.4 (4)
C3—C4—H4	108.9	O2—C8—C81	108.5 (4)
O5—C5—C6	102.8 (4)	O1—C8—C81	108.6 (4)
O5—C5—C4	108.1 (3)	C82—C8—C81	113.3 (5)
C6—C5—C4	115.4 (4)	C8—C81—H81A	109.5
O5—C5—H5	110.1	C8—C81—H81B	109.5
C6—C5—H5	110.1	H81A—C81—H81B	109.5
C4—C5—H5	110.1	C8—C81—H81C	109.5
O6—C6—C5	103.3 (5)	H81A—C81—H81C	109.5
O6—C6—H6A	111.1	H81B—C81—H81C	109.5
C5—C6—H6A	111.1	C8—C82—H82A	109.5
O6—C6—H6B	111.1	C8—C82—H82C	109.5
C5—C6—H6B	111.1	H82A—C82—H82C	109.5
H6A—C6—H6B	109.1	C8—C82—H82B	109.5
O6—C7—O5	106.7 (4)	H82A—C82—H82B	109.5
O6—C7—C72A	105.2 (14)	H82C—C82—H82B	109.5
O5—C7—C72A	110.0 (8)	C3—C9—H9C	109.5
O6—C7—C71A	107.4 (16)	C3—C9—H9B	109.5
O5—C7—C71A	107.9 (12)	H9C—C9—H9B	109.5
C72A—C7—C71A	118.9 (19)	C3—C9—H9A	109.5
O6—C7—C71B	106 (5)	H9C—C9—H9A	109.5
O5—C7—C71B	119 (4)	H9B—C9—H9A	109.5
O6—C7—C72B	127 (3)		
			
C8—O1—C1—O4	−111.3 (4)	C7—O5—C5—C4	−153.6 (4)
C8—O1—C1—C2	4.3 (5)	O4—C4—C5—O5	171.2 (4)
C4—O4—C1—O1	100.8 (3)	C3—C4—C5—O5	−71.9 (4)
C4—O4—C1—C2	−13.8 (4)	O4—C4—C5—C6	56.8 (5)
C8—O2—C2—C1	24.8 (4)	C3—C4—C5—C6	173.7 (4)
C8—O2—C2—C3	136.1 (3)	C7—O6—C6—C5	−21.4 (7)
O1—C1—C2—O2	−17.4 (4)	O5—C5—C6—O6	31.7 (6)
O4—C1—C2—O2	101.6 (3)	C4—C5—C6—O6	149.1 (4)
O1—C1—C2—C3	−130.8 (3)	C6—O6—C7—O5	2.5 (7)
O4—C1—C2—C3	−11.8 (4)	C6—O6—C7—C72A	−114.4 (10)
O2—C2—C3—O3	39.9 (4)	C6—O6—C7—C71A	118.0 (13)
C1—C2—C3—O3	150.4 (3)	C6—O6—C7—C71B	130 (3)
O2—C2—C3—C9	160.6 (3)	C6—O6—C7—C72B	−117 (3)
C1—C2—C3—C9	−88.8 (4)	C5—O5—C7—O6	18.7 (6)
O2—C2—C3—C4	−80.2 (3)	C5—O5—C7—C72A	132.4 (15)
C1—C2—C3—C4	30.3 (4)	C5—O5—C7—C71A	−96.4 (17)
C1—O4—C4—C5	160.1 (3)	C5—O5—C7—C71B	−101 (5)
C1—O4—C4—C3	33.7 (4)	C5—O5—C7—C72B	154 (3)
O3—C3—C4—O4	−158.5 (3)	C2—O2—C8—O1	−22.4 (5)
C9—C3—C4—O4	78.9 (4)	C2—O2—C8—C82	96.6 (5)
C2—C3—C4—O4	−38.6 (3)	C2—O2—C8—C81	−138.5 (4)
O3—C3—C4—C5	82.7 (4)	C1—O1—C8—O2	10.6 (5)
C9—C3—C4—C5	−39.8 (4)	C1—O1—C8—C82	−108.5 (5)
C2—C3—C4—C5	−157.4 (3)	C1—O1—C8—C81	126.6 (4)
C7—O5—C5—C6	−31.1 (6)		

### Hydrogen-bond geometry (Å, º)

**Table:**

*D*—H···*A*	*D*—H	H···*A*	*D*···*A*	*D*—H···*A*
O3—H3···O4^i^	0.82	2.28	3.022 (4)	152
C1—H1···O2^ii^	0.98	2.58	3.218 (5)	123
C2—H2···O6^iii^	0.98	2.54	3.504 (5)	167

Symmetry codes: (i) *x*−1, *y*, *z*; (ii) *x*+1, *y*, *z*; (iii) *x*, *y*, *z*+1.
